# Association between *Clonorchis sinensis* infection and the risk of pulmonary fungal infection in patients with AECOPD: a retrospective propensity score matching study

**DOI:** 10.3389/fimmu.2026.1913508

**Published:** 2026-08-10

**Authors:** Qinglan Li, Hanlin Liang, Yuanlan Wu, Luxi Lei, Hongchun Huang, Zhixiong He, Siyu Lei, Shiya Lin, Yinying Li, Zhaoqin Jiang, Minchao Duan

**Affiliations:** 1 Department of Respiratory and Critical Care Medicine, Wuming Hospital of Guangxi Medical University, Nanning, China; 2 Guangxi Medical University, Nanning, China

**Keywords:** chronic obstructive pulmonary disease, *Clonorchis sinensis*, immune regulation, lymphocytes, mediation analysis, pulmonary fungal infection

## Abstract

**Background:**

Pulmonary fungal infection (PFI) is a severe complication in patients with acute exacerbations of chronic obstructive pulmonary disease (AECOPD). Although helminth infections are increasingly recognized as potent modulators of host immunity, their influence on susceptibility to opportunistic fungal infections remains poorly understood. We investigated whether Clonorchis sinensis (*C. sinensis*) infection is associated with risk of PFI in AECOPD patients and explored potential immunological mechanisms underlying this association.

**Methods:**

We conducted a retrospective cohort study including 2,523 patients hospitalized for AECOPD between January 2018 and December 2025. Following 1:4 propensity score matching (PSM), 217 patients with *C. sinensis* infection were matched to 840 controls. The primary outcome was in-hospital PFI. A five-tier causal inference framework incorporating PSM, multivariable conditional logistic regression, inverse probability of treatment weighting (IPTW), and doubly robust estimation was used to evaluate robustness of the association. Robustness was validated through sensitivity analyses adjusting for acute exacerbation severity (arterial blood gas parameters) and applying strictly defined PFI criteria. Causal mediation analyses assessed contributions from peripheral immune cells and clinical treatments.

**Results:**

Post-matching, the *C. sinensis* group exhibited a significantly lower PFI incidence than controls (5.1% vs. 10.5%, *P* = 0.021). This association remained consistent across all primary causal inference models (Odds Ratio [OR] range: 0.455–0.465, all *P* < 0.05). These findings were further confirmed by sensitivity analyses adjusting for acute exacerbation severity (OR = 0.448, *P* = 0.018) and restricting the outcome to strictly defined PFI cases receiving ≥ 7 days of systemic antifungal therapy (OR = 0.376, *P* = 0.016; E-value = 4.76). Mediation analyses revealed that the quantitative preservation of peripheral absolute lymphocyte counts accounted for 12.11% of this association, while a reduced duration of systemic corticosteroids exposure mediated an additional 10.91%.

**Conclusions:**

*C. sinensis* infection is independently associated with a substantially lower risk of PFI in hospitalized AECOPD patients. This association is partially mediated by two parallel pathways: the quantitative preservation of peripheral lymphocyte counts and a reduced duration of systemic corticosteroids exposure. These findings provide novel clinical evidence linking helminth-associated immune remodeling to altered host susceptibility to opportunistic infections.

## 1 Introduction

Chronic obstructive pulmonary disease (COPD) is a major global health challenge and remains one of the leading causes of morbidity and mortality worldwide (1, 2). Acute exacerbations of COPD (AECOPD) accelerate lung function decline, worsen quality of life, and substantially increase healthcare utilization and mortality (3, 4). In addition to bacterial and viral pathogens, opportunistic fungal infections have emerged as an increasingly important complication among patients hospitalized for AECOPD (5). Structural airway damage, impaired mucosal barrier function, repeated exposure to broad-spectrum antibiotics, and corticosteroids-induced immunosuppression collectively disrupt host antifungal defense and raise susceptibility to pulmonary fungal infections (PFI) (6). The development of PFI in patients with AECOPD is associated with prolonged hospitalization, increased healthcare costs, and unfavorable clinical outcomes (7, 8). This clinical background highlights the vital role of host immune function in shaping fungal infection risk.

Host immune status is a critical determinant of whether inhaled fungal organisms remain harmless colonizers or progress to clinically significant infections (9). Effective antifungal immunity requires coordinated interactions between innate and adaptive immune responses. Neutrophils, macrophages, and dendritic cells provide the first line of defense against fungal pathogens, whereas adaptive immune responses, particularly CD4+ T cell subsets, play essential roles in maintaining mucosal barrier integrity and orchestrating fungal clearance (10–12). Accordingly, loss of balanced adaptive immune function is recognized as a key risk factor that renders COPD patients vulnerable to invasive fungal complications (6, 13).

Beyond their direct pathogenic effects, helminth infections are increasingly recognized as potent regulators of host immunity. Through long-term co-evolution with their hosts, helminths have developed sophisticated strategies to regulate immune responses and promote persistent survival (14, 15). Experimental and epidemiological studies have demonstrated that helminth infections can induce profound immunological remodeling characterized by expansion of regulatory T cells (Tregs), increased production of anti-inflammatory cytokines such as interleukin-10 (IL-10) and transforming growth factor-β (TGF-β), and dynamic alterations in T helper cell polarization (16–18). These immunoregulatory effects have been implicated in the modulation of allergic diseases, autoimmune disorders, and chronic inflammatory conditions (19, 20). Given the central role of host immune status in determining susceptibility to opportunistic pathogens, helminth-associated immune regulation may also influence susceptibility to secondary infections. However, whether chronic helminth infection affects the risk of opportunistic fungal infections remains poorly understood.

*Clonorchis sinensis (C. sinensis)* is a food-borne liver fluke endemic to East and Southeast Asia and remains highly prevalent in southern China (21, 22). Although traditionally regarded as a hepatobiliary parasite, accumulating evidence indicates that *C. sinensis* infection exerts systemic immunological effects extending beyond the hepatobiliary tract (23–25). Because COPD and clonorchiasis frequently coexist in endemic regions, *C. sinensis* infection may represent a previously underrecognized factor influencing susceptibility to pulmonary fungal infections in patients with AECOPD. Nevertheless, no clinical study has investigated whether *C. sinensis* infection modulates the risk of pulmonary fungal infections in patients with AECOPD, a population with impaired antifungal immunity and high exposure to immunosuppressive therapies.

Given the central role of adaptive immunity in antifungal defense and the established immunomodulatory properties of helminth infection, we hypothesized that *C. sinensis* infection may influence susceptibility to pulmonary fungal infections in patients with AECOPD. Therefore, we conducted a retrospective propensity score-matched cohort study to investigate the association between *C. sinensis* infection and the risk of PFI among hospitalized patients with AECOPD. Furthermore, by integrating multiple causal inference approaches and mediation analyses, we sought to explore potential immunological and clinical pathways underlying this association. Understanding how helminth-associated immune regulation shapes susceptibility to opportunistic fungal infections may provide new insights into host–parasite–microbe interactions and identify novel targets for host-directed antifungal therapies.

## 2 Methods

### 2.1 Study design and participants

**Figure 1:**
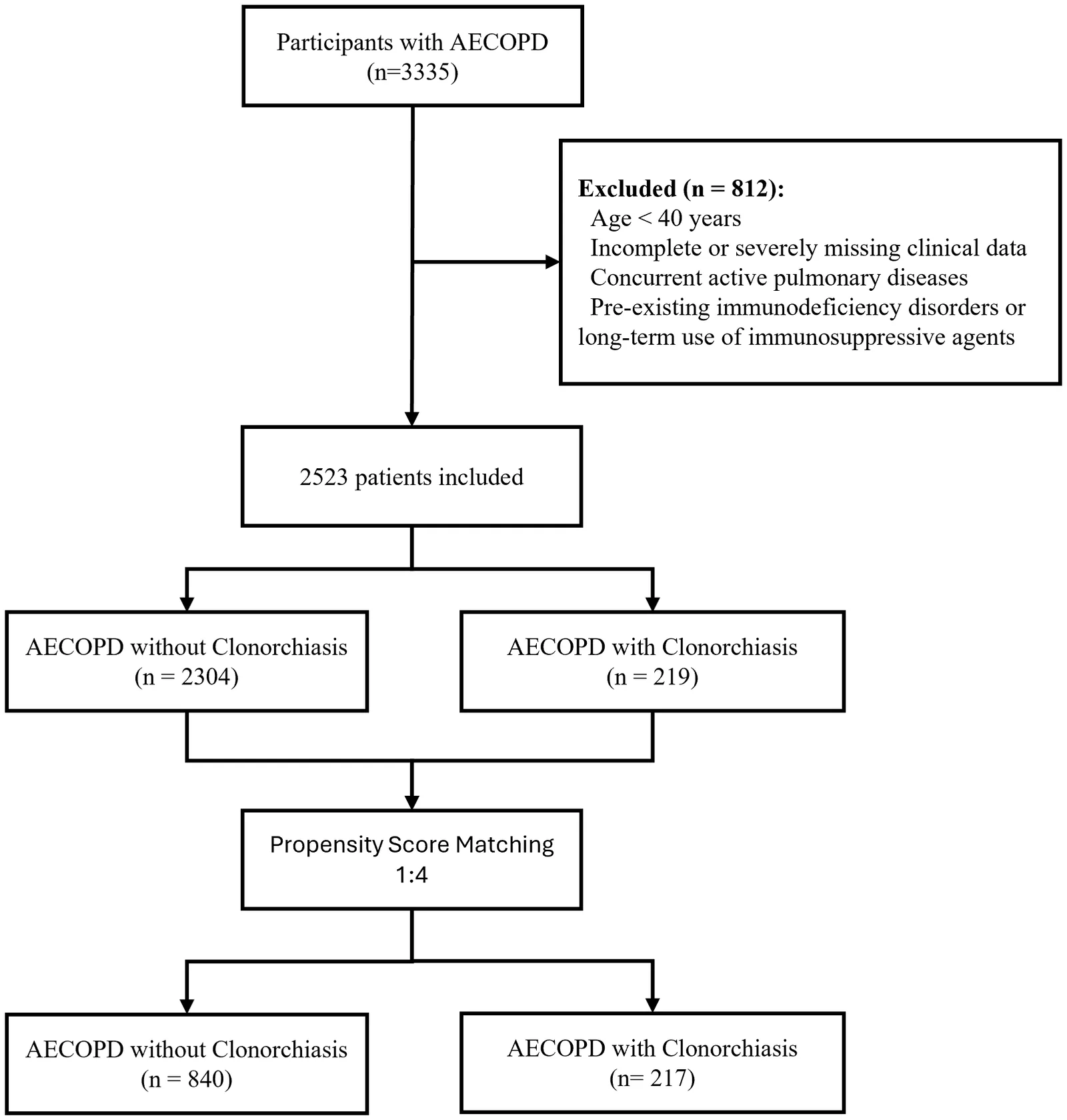
Flowchart of study participant selection and propensity score matching.

This retrospective observational cohort study was designed to investigate the relationship between *Clonorchis sinensis* infection, host immune status, and susceptibility to PFI in patients hospitalized for AECOPD. Electronic medical records were reviewed for consecutive patients admitted to Wuming Hospital of Guangxi Medical University between January 2018 and December 2025. The study protocol was approved by Wuming Hospital of Guangxi Medical University Medical Ethical Review Committee. Owing to the retrospective design and the use of anonymized clinical data, the requirement for written informed consent was waived. Eligible participants were adults aged ≥40 years with a confirmed diagnosis of AECOPD according to the Global Initiative for Chronic Obstructive Lung Disease (GOLD) criteria. Patients were excluded if they had severe missing clinical records, concomitant pulmonary diseases other than COPD (including asthma, tuberculosis, bronchiectasis, lung cancer and interstitial lung disease), known immunodeficiency disorders, or long-term (≥ 3weeks) treatment with immunosuppressive agents. The detailed patient selection process is presented in **Figure 1**. After application of all inclusion and exclusion criteria, 2,523 eligible patients were included in the initial cohort, comprising 219 patients with *C. sinensis* infection and 2,304 uninfected controls.

### 2.2 Clinical data extraction

Baseline demographic and clinical data were extracted from the electronic medical record system, including age, sex, body mass index (BMI), smoking history, alcohol consumption, pulmonary function measured by forced expiratory volume in one second as a percentage of the predicted value (FEV1% predicted), and comorbidities (hypertension, diabetes mellitus, coronary heart disease, hypoalbuminemia, and hepatic impairment). Hepatic impairment was strictly defined as the presence of clear precipitating factors accompanied by elevations of total bilirubin or alanine aminotransferase (ALT) above the upper limit of normal. Laboratory and physiological assessments performed at hospital admission included complete blood counts, differential leukocyte counts, liver function indicators, and arterial blood gas analyses (including pH, PaO_2_, PaCO_2_, and the PaO_2_/FiO_2_ ratio). Information regarding in-hospital therapeutic interventions was also collected, including systemic corticosteroids therapy, inhaled corticosteroid (ICS) use, reserve antibiotic administration, and respiratory support. Reserve antibiotics were defined as systemic administration of carbapenems, tigecycline, vancomycin, linezolid, or polymyxins. The primary clinical outcome was the occurrence of PFI during hospitalization. Secondary outcomes included length of hospital stay, intensive care unit (ICU) admission, non-invasive ventilation (NIV), and invasive mechanical ventilation (IMV).

### 2.3 Definition of *Clonorchis sinensis* infection

Diagnosis of *C. sinensis* infection was determined according to the Chinese National Standard for Clonorchiasis (WS309–2009). Patients were classified as having clonorchiasis if they met either etiological or clinical diagnostic criteria. Etiological confirmation, considered the diagnostic gold standard, required direct identification of *Clonorchis sinensis* eggs in stool specimens or bile drainage fluid. Clinical diagnosis required a documented diagnosis of clonorchiasis together with either a compatible epidemiological history (consumption of raw or undercooked freshwater fish within the previous five years) or characteristic biliary imaging findings, including diffuse intrahepatic bile duct dilatation without evidence of mechanical obstruction on ultrasonography, CT, or MRI.

### 2.4 Definition of pulmonary fungal infection

The primary clinical outcome of this study is PFI occurring during hospitalization for AECOPD. PFI was defined by the presence of (1) Clinical and radiological deterioration compatible with fungal infection: including persistent fever despite ≥3 days of broad-spectrum antibiotic therapy, new or progressive pulmonary infiltrates on chest imaging, or worsening respiratory symptoms/hypoxemia that could not be explained by bacterial or viral pathogens;(2) microbiological evidence from qualified lower respiratory tract samples (deep sputum, endotracheal aspirates, or BALF), defined as either positive fungal culture, BALF galactomannan (ODI ≥ 1.0), or positive metagenomic next-generation sequencing (mNGS) results, and (3) initiation of systemic antifungal therapy during hospitalization based on integrated clinical, radiological, and microbiological findings. Isolation of fungi from respiratory specimens in the absence of compatible clinical deterioration was classified as airway colonization and was not counted as PFI. Antifungal therapy was defined as systemic antifungal treatment started during hospitalization for suspected or confirmed fungal infection.

### 2.5 Assessment of systemic immune phenotypes

To characterize systemic immune phenotypes associated with *C. sinensis* infection, peripheral blood immune cell populations and composite immune-inflammatory indices were evaluated using complete blood count data obtained at hospital admission. A high eosinophil phenotype was defined as an absolute peripheral blood eosinophil count > 0.3 × 10^9^/L. All variables utilized in the calculations represent absolute cell counts (expressed as 10^9^/L). The definitions and specific calculation formulas are detailed as follows: Neutrophil-to-Lymphocyte Ratio (NLR): NLR = Absolute Neutrophil Count/Absolute Lymphocyte Count. Platelet-to-Lymphocyte Ratio (PLR): PLR = Absolute Platelet Count/Absolute Lymphocyte Count. Eosinophil-to-Lymphocyte Ratio (ELR): ELR = Absolute Eosinophil Count/Absolute Lymphocyte Count. Systemic Immune-Inflammation Index (SII): SII = (Absolute Platelet Count × Absolute Neutrophil Count)/Absolute Lymphocyte Count. These parameters were selected to capture distinct dimensions of innate immunity, adaptive immunity, and systemic inflammatory status.

### 2.6 Statistical analysis

Categorical variables were expressed as frequencies and percentages and compared using the chi-square or Fisher’s exact test. Continuous variables were tested for normality using the Shapiro-Wilk test; normally distributed data were presented as mean ± standard deviation (SD) and analyzed via Student’s t-test, while non-normally distributed data were expressed as medians with interquartile ranges (IQR) and compared using the Mann-Whitney U test. Missing data in baseline covariates were handled using multiple imputation by chained equations (MICE) with 10 imputed datasets generated. The imputation model covered all matching and adjustment covariates. The primary outcome was not imputed to avoid potential bias. Primary analyses (PSM, regression, E-value and sensitivity analyses) adopted pooled imputed datasets; laboratory immune and blood gas indicators were analyzed with complete cases to guarantee measurement accuracy. To minimize baseline confounding in this hospitalized cohort, 1:4 propensity score matching (PSM) was performed using nearest-neighbor matching with a caliper of 0.1. Covariates included demographics, lifestyle factors, pulmonary function (FEV1% predicted), and comorbidities (hypertension, diabetes, coronary heart disease, hypoalbuminemia, and hepatic impairment). Balance was verified by standardized mean difference (SMD), with SMD < 0.1 indicating excellent balance. To evaluate the robustness of the association between *C. sinensis* infection and PFI, we established a hierarchical causal inference framework. Model 1 comprised an unadjusted analysis within the matched cohort. Model 2 applied multivariable conditional logistic regression adjusted for post-matching baseline characteristics. Model 3 further incorporated treatment-related confounders, including systemic/inhaled corticosteroids, reserve antibiotics, and invasive mechanical ventilation. Model 4 utilized stabilized inverse probability of treatment weighting (IPTW), and Model 5 employed a doubly robust estimation integrating IPTW and multivariable regression. Potential unmeasured confounding was quantified using E-values. To assess the robustness of our conclusions against specific methodological challenges, we performed two sensitivity analyses based on Model 2: (1) to account for potential confounding by acute disease severity, we conducted an exploratory regression adjusted for arterial blood gas parameters (PaO_2_/FiO_2_ ratio and PaCO_2_); and (2) to minimize diagnostic bias from potential airway colonization, we restricted the outcome to strict PFI cases defined by a full course of systemic antifungal therapy (≥ 7 days). Causal mediation analyses were conducted to explore the underlying biological and clinical pathways. To ensure temporal precedence, all candidate mediators, including absolute counts of lymphocytes, neutrophils, and eosinophils, and the duration of systemic corticosteroids exposure, were ascertained prior to the clinical diagnosis of PFI or the initiation of systemic antifungal therapy. The average causal mediation effect (ACME) and the proportion of mediation were calculated. Statistical analyses were performed using R (version 4.5.2), with two-sided P values < 0.05 considered statistically significant.

## 3 Results

### 3.1 Study population and propensity score matching

The patient screening and enrollment workflow is illustrated in **Figure 1**. Initially, a total of 3,335 patients hospitalized for AECOPD were screened for eligibility. Following the rigorous application of the inclusion and exclusion criteria, 2,304 patients were assigned to the control group and 219 to the *C. sinensis* group. As detailed in the left panel of **Table 1**, the pre-matched cohorts exhibited significant heterogeneity across several baseline characteristics. Specifically, compared to the control group, patients in the *C. sinensis* group were significantly younger, comprised a higher proportion of males, had higher rates of smoking, and showed a lower prevalence of hypoalbuminemia (all *P* < 0.05).

**Table 1:** Baseline characteristics of the study population before and after propensity score matching.

Variables	Before PSM			After PSM			
	Control (n = 2304)	*C. sinensis* (n = 219)	p-Value	Control (n = 840)	*C. sinensis* (n = 217)	p-Value	SMD
Demographics							
Age, years	73.48 (9.77)	70.25 (10.46)	<0.001	70.48 (10.00)	70.34 (10.46)	0.852	0.014
Male sex, n (%)	1860 (80.7)	200 (91.3)	<0.001	744 (88.6)	198 (91.2)	0.315	0.089
BMI, kg/m^2^	20.81 [18.36, 23.44]	20.31 [18.39, 22.92]	0.724	20.76 [18.36, 23.44]	20.31 [18.37, 22.89]	0.732	0.023
Clinical parameter							
FEV1 (% predicted)	43.40 [31.20, 61.50]	44.30 [33.30, 66.40]	0.232	44.30 [31.17, 64.20]	44.20 [33.30, 65.50]	0.472	0.012
Lifestyle							
Smoking status, n (%)			<0.001			0.878	0.039
Current smoker	353 (15.3)	51 (23.3)		184 (21.9)	50 (23.0)		
Former smoker	862 (37.4)	94 (42.9)		355 (42.3)	93 (42.9)		
Never smoked	1089 (47.3)	74 (33.8)		301 (35.8)	74 (34.1)		
Drinkers, n (%)	574 (24.9)	66 (30.1)	0.106	245 (29.2)	66 (30.4)	0.782	0.027
Comorbidities							
Hypertension, n (%)	874 (37.9)	78 (35.6)	0.546	298 (35.5)	78 (35.9)	0.961	0.010
Diabetes, n (%)	161 (7.0)	11 (5.0)	0.336	51 (6.1)	11 (5.1)	0.691	0.044
Coronary heart disease, n (%)	753 (32.7)	60 (27.4)	0.128	215 (25.6)	60 (27.6)	0.597	0.046
Hypoalbuminemia, n (%)	541 (23.5)	38 (17.4)	0.048	144 (17.1)	38 (17.5)	0.978	0.010
Hepatic impairment, n (%)	149 (6.5)	13 (5.9)	0.871	57 (6.8)	13 (6.0)	0.790	0.033
Total bilirubin, μmol/L	9.15 [6.58, 13.53]	8.93 [6.66, 12.88]	0.59	9.28 [6.74, 13.83]	8.93 [6.66, 12.88]	0.378	0.028
ALT, U/L	16.00 [10.80, 23.00]	16.00 [11.00, 24.00]	0.462	16.30 [11.00, 24.70]	16.00 [11.00, 24.00]	0.661	0.087
AST, U/L	22.00 [17.00, 30.00]	23.00 [18.00, 28.00]	0.463	22.00 [17.40, 31.00]	23.00 [18.00, 28.00]	0.805	0.028

ALT, Alanine aminotransferase; AST, Aspartate aminotransferase; BMI, body mass index; *C. sinensis*, *Clonorchis sinensis*; FEV1, forced expiratory volume in 1 second; PSM, propensity score matching; SMD, standardized mean difference.

Given the observational design of this study, a 1:4 PSM protocol was implemented to maximally mitigate the influence of potential baseline confounders on clinical outcomes. Post-matching, the core analysis cohort comprised 840 control patients and 217 C*. sinensis* patients. As demonstrated in the right panel of **Table 1**, PSM effectively balanced all predefined baseline covariates between the two groups. These encompassed demographics (age, sex, BMI), clinical parameters (FEV1% predicted), lifestyle factors (smoking and alcohol consumption), and a comprehensive set of comorbidities including hypertension, diabetes, coronary heart disease, hypoalbuminemia, and hepatic impairment (with specific liver function indicators: total bilirubin, ALT, and AST). All post-matching SMDs were well below 0.1, and all *P*-values were > 0.05. Therefore, PSM effectively minimized baseline confounding, yielding a highly comparable cohort for the subsequent analysis of clinical outcomes.

### 3.2 Alterations in systemic immune and inflammatory status

We evaluated the baseline immunological profiles of the matched cohort. As summarized in **Table 2**, the *C. sinensis* group exhibited a distinct systemic immune signature. Compared to the control group, patients in the *C. sinensis* group demonstrated significantly elevated absolute counts of peripheral blood lymphocytes (*P* = 0.003), as well as significantly higher absolute counts and relative percentages of eosinophils (*P* = 0.004 and *P* = 0.012, respectively). The relative percentage of lymphocytes did not reach statistical significance (*P* = 0.096), and the proportion of neutrophils only displayed a decreasing trend rather than a formally significant reduction (*P* = 0.057). Notably, the prevalence of the high eosinophil phenotype was substantially greater in the *C. sinensis* group (35.9% vs. 24.6%, *P* = 0.001).

**Table 2:** Immune and inflammatory characteristics after propensity score matching.

Variables	Control (n = 840)	*C. sinensis* (n = 217)	p-Value
Leukocyte composition			
WBC, 10^9^/L	8.00 [6.13, 10.53]	8.32 [6.25, 11.44]	0.088
Neutrophils, %	74.45 [64.60, 82.80]	71.70 [60.80, 82.90]	0.057
Neutrophils, 10^9^/L	5.73 [4.12, 8.40]	5.49 [3.97, 9.07]	0.917
Lymphocytes, %	14.50 [8.50, 21.20]	16.30 [9.40, 23.70]	0.096
Lymphocytes, 10^9^/L	1.11 [0.73, 1.61]	1.29 [0.79, 1.81]	0.003
Eosinophils, %	1.55 [0.20, 4.00]	2.30 [0.30, 6.40]	0.012
Eosinophils, 10^9^/L	0.12 [0.02, 0.29]	0.19 [0.02, 0.47]	0.004
Inflammatory markers			
CRP, mg/L	10.84 [3.14, 44.78]	10.30 [2.75, 46.23]	0.877
Immune indices			
NLR	5.12 [3.13, 9.55]	4.24 [2.44, 8.86]	0.034
ELR	0.10 [0.02, 0.25]	0.13 [0.02, 0.35]	0.065
SII	1271.23 [671.23, 2393.10]	1111.23 [575.17, 2497.78]	0.272
PLR	215.92 [140.33, 350.16]	196.00 [126.25, 325.00]	0.108
Immune phenotype			
High eosinophils, n (%)	207 (24.6)	78 (35.9)	0.001

WBC, white blood cell; CRP, C-reactive protein; *C. sinensis*, *Clonorchis sinensis*; NLR, neutrophil-to-lymphocyte ratio; ELR, eosinophil-to-lymphocyte ratio; SII, systemic immune-inflammation index; PLR, platelet-to-lymphocyte ratio. Differential leukocyte counts are presented as both percentages (%) and absolute counts (10^9^/L). High eosinophils was defined as an eosinophil count >0.3 × 10^9^/L.

Correspondingly, significant deviations were observed in specific composite systemic immune indices. The NLR was significantly lower in the *C. sinensis* group (*P* = 0.034). Other indices, including the ELR (*P* = 0.065), PLR (*P* = 0.108), and SII (*P* = 0.272), did not demonstrate statistically significant differences. These findings suggest that *C. sinensis* infection in this hospitalized cohort is associated with a specific peripheral immune profile characterized primarily by elevated absolute lymphocyte counts and a prominent eosinophilic response.

### 3.3 Reduction in pulmonary fungal infection

As detailed in **Table 3**, the overall incidence of PFI was significantly lower in the *C. sinensis* group compared with the control group (5.1% vs. 10.5%, *P* = 0.021). In pathogen-specific analyses, this overall reduction was primarily driven by a significant decline in *Candida* spp. infections, which decreased from 5.83% in the control group to 1.84% in the *C. sinensis* group (*P* = 0.017). The incidence of *Aspergillus* spp. infections remained comparable between the cohorts (2.30% vs. 2.62%, *P* = 0.817). Crucially, when restricting the mycological spectrum analysis solely to patients with confirmed fungal infections, the proportional distribution of *Aspergillus* and *Candida* species showed no significant difference between the two groups (all *P* > 0.05). These data indicate a reduction in overall host susceptibility to fungal infections in the *C. sinensis* group, rather than a shift in the mycological distribution among infected patients.

**Table 3:** In-hospital clinical outcomes after propensity score matching.

Variables	Control (n = 840)	*C. sinensis* (n = 217)	p-Value
Primary outcome			
Pulmonary fungal infection, n (%)	88 (10.5)	11 (5.1)	0.021
Strict PFI (antifungal therapy ≥ 7 days), n (%)	73 (8.7%)	8 (3.7%)	0.017
Pathogen-specific incidence in the overall cohort			
*Aspergillus* spp., n (%)	22 (2.62%)	5 (2.30%)	0.817
*Candida* spp. (total), n (%)	49 (5.83%)	4 (1.84%)	0.017
Other fungi, n (%)	17 (2.02%)	2 (0.92%)	0.399
Fungal infection spectrum among infected patients			
*Aspergillus* spp., n (%)	22 (25.00%)	5 (45.45%)	0.170
*Candida* spp. (total), n (%)	49 (55.68%)	4 (36.36%)	0.320
Other fungi, n (%)	17 (19.32%)	2 (18.18%)	1.000
Acute physiological outcomes at exacerbation			
pH	7.42 [7.38, 7.44]	7.43 [7.40, 7.45]	0.004
PaO_2_ (mmHg)	82.00 [69.00, 98.00]	83.00 [70.40, 100.00]	0.472
PaO_2_/FiO_2_ ratio	306.90 [251.72, 375.94]	321.43 [262.07, 365.52]	0.255
PaCO_2_ (mmHg)	41.00 [36.88, 49.00]	40.00 [36.40, 44.00]	0.026
Clinical outcomes and in-hospital interventions			
ICU admission, n (%)	88 (10.5)	19 (8.8)	0.533
Length of stay (days)	9.00 [6.00, 12.00]	9.00 [6.00, 12.00]	0.739
Systemic corticosteroids use, n (%)	496 (59.0)	111 (51.2)	0.043
Duration of systemic corticosteroids (days)	2.27 (3.24)	1.73 (2.53)	0.023
Reserve antibiotics use, n (%)	41 (4.9)	9 (4.1)	0.784
Respiratory failure, n (%)	197 (23.5)	39 (18.0)	0.102
NIV use, n (%)	74 (8.8)	10 (4.6)	0.058
IMV use, n (%)	51 (6.1)	10 (4.6)	0.509

*C. sinensis*, *Clonorchis sinensis*; ICU, intensive care unit; NIV, non-invasive ventilation; IMV, invasive mechanical ventilation; spp., species pluralis.

### 3.4 Clinical outcomes and interventions

Beyond the primary outcome, we evaluated the impact of *C. sinensis* infection on secondary clinical outcomes during hospitalization among patients with AECOPD (**Table 3**). Regarding clinical pharmacotherapy, both the administration frequency (51.2% vs. 59.0%, *P* = 0.043) and the duration (*P* = 0.023) of systemic corticosteroids therapy were significantly reduced in the *C. sinensis* group compared to the control group. The utilization of reserve antibiotics showed no statistically significant difference between the two groups (*P* = 0.784).

Regarding overall hospitalization characteristics and respiratory support requirements, both groups had an identical median length of stay of 9 days (*P* = 0.739). Concurrently, the two cohorts exhibited similar rates of intensive care unit (ICU) admission (8.8% vs. 10.5%, *P* = 0.533) and invasive mechanical ventilation (IMV) use (4.6% vs. 6.1%, *P* = 0.509). During the exacerbation, the incidence of respiratory failure showed a downward trend in the infected group (18.0% vs. 23.5%, *P* = 0.102). Similarly, the requirement for non-invasive ventilation (NIV) displayed a trending decrease in the *C. sinensis* group (4.6% vs. 8.8%, *P* = 0.058). Physiologically, although the *C. sinensis* group exhibited a higher blood pH and lower PaCO_2_, core oxygenation parameters, including PaO_2_ and the PaO_2_/FiO_2_ ratio, remained comparable between the two cohorts (*P* = 0.472 and *P* = 0.255, respectively). Collectively, these results demonstrate that *C. sinensis* infection in hospitalized AECOPD patients is associated with a distinct clinical trajectory, characterized by a trending reduction in the need for non-invasive respiratory support and significantly lower systemic corticosteroids exposure, without altering the overall hospital course.

### 3.5 Robustness validation via multiple causal inference models

**Figure 2:**
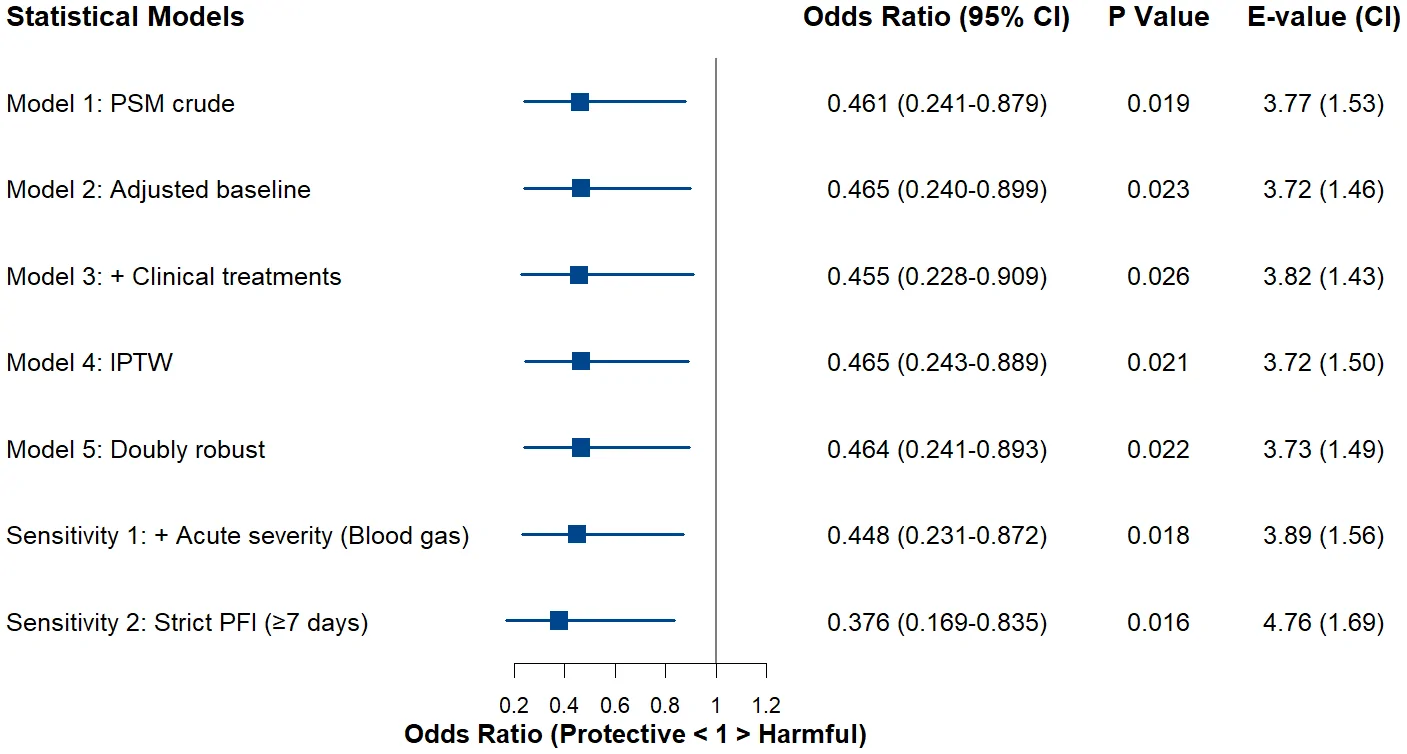
Forest plot of the association between *C. sinensis* infection and a reduced risk of pulmonary fungal infection across five distinct statistical models. Model 1 (PSM crude): Conditional logistic regression based on the propensity score-matched cohort. Model 2 (Adjusted baseline): Multivariable conditional logistic regression adjusted for age, gender, BMI, smoking status, FEV1% predicted, diabetes, hypoalbuminemia and hepatic impairment. Model 3 (+ Clinical treatments): Further adjustment for clinical treatments, including systemic corticosteroids, inhaled corticosteroids (ICS), reserve antibiotics, and invasive mechanical ventilation (IMV). Model 4 (IPTW): Analysis using Stabilized Inverse Probability of Treatment Weighting to minimize selection bias. Model 5 (Doubly robust): A combined approach integrating both IPTW and multivariable regression for the most conservative and robust estimate. Sensitivity analysis: Sensitivity 1 adjusted for arterial blood gas parameters (PaO_2_/FiO_2_ ratio and PaCO_2_) to account for acute exacerbation severity; Sensitivity 2 evaluated this association by restricting the outcome to strict PFI (defined as receiving systemic antifungal therapy for ≥ 7 days). E-values were calculated to quantify the potential impact of unmeasured confounding.

To ensure that the observed association did not stem from statistical bias or residual confounding, multiple advanced causal inference frameworks and dedicated sensitivity analyses were conducted (**Figure 2**). The unadjusted analysis within the PSM cohort (Model 1: PSM crude) initially revealed a substantial association of *C. sinensis* infection against PFI (odds ratio [OR] = 0.461; 95% confidence interval [CI]: 0.241–0.879; *P* = 0.019). This association demonstrated remarkable stability across progressively stringent statistical adjustments and alternative weighting methods (Models 2 through 5), with the estimated ORs converging narrowly between 0.455 and 0.465 (all *P* < 0.05).

Furthermore, sensitivity analyses were conducted to evaluate the robustness of these findings against specific methodological challenges. Even when accounting for potential unmeasured confounding from acute disease severity via blood gas parameters (Sensitivity 1), the association remained significant (OR = 0.448; 95% CI: 0.231–0.872; *P* = 0.018). Similarly, when restricting the outcome to strictly defined PFI cases receiving systemic antifungal therapy for ≥ 7 days to minimize diagnostic bias (Sensitivity 2), this association with a reduced risk was substantially reinforced (OR = 0.376; 95% CI: 0.169–0.835; *P* = 0.016). To formally quantify the potential impact of unmeasured confounding, E-values were calculated, reaching up to 4.76 for the strict PFI sensitivity analysis. This indicates that an unmeasured confounder would need to be associated with both *C. sinensis* infection and strict PFI by a risk ratio of at least 4.76 to completely explain away this association. The consistency of effect estimates and the highly robust E-values further solidify the reliability of our findings.

### 3.6 Mediation analysis of peripheral immune cell counts and systemic corticosteroid exposure

**Figure 3:**
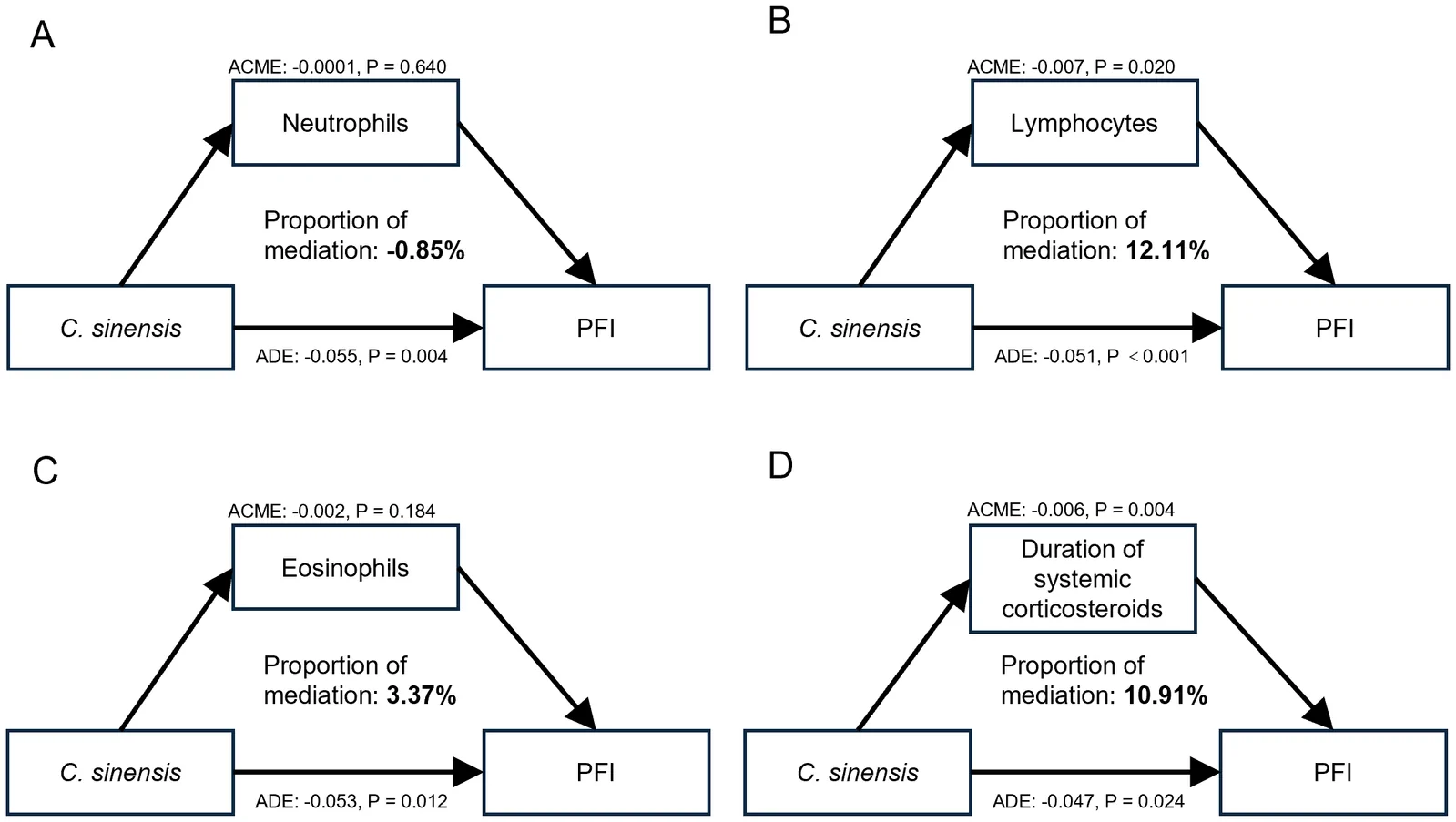
Mediation analysis of immune cell counts and systemic corticosteroids duration. **(A–D)** The mediation pathways through which *C. sinensis* infection influences the risk of pulmonary fungal infections (PFI) via **(A)** absolute neutrophil count, **(B)** absolute lymphocyte count, **(C)** absolute eosinophil count, and **(D)** duration of systemic corticosteroids. PFI, pulmonary fungal infection; Abs, absolute count; ACME, average causal mediation effect; ADE, average direct effect.

A mediation analysis was conducted to explore the potential pathways linking *C. sinensis* infection to a reduced risk of PFI (**Figure 3**). Among the peripheral blood immune parameters, the absolute lymphocyte count emerged as a significant mediator (ACME = -0.007, *P* = 0.020; **Figure 3B**), accounting for 12.11% of this overall association. Conversely, despite the baseline elevation of eosinophils in the *C. sinensis* group, the absolute eosinophil count did not significantly mediate this relationship (proportion mediated = 3.37%, ACME = -0.002, *P* = 0.184; **Figure 3C**). Similarly, the absolute neutrophil count showed no mediating role (proportion mediated = -0.85%, ACME = -0.0001, *P* = 0.640; **Figure 3A**). These findings suggest that the elevation of the peripheral absolute lymphocyte count may partially explain the association between *C. sinensis* infection and reduced fungal susceptibility, rather than being driven by nonspecific granulocytic responses. Furthermore, we evaluated clinical treatment differences as potential mediators. The duration of systemic corticosteroids therapy was identified as a significant clinical mediator, explaining 10.91% of the association (ACME = -0.006, *P* = 0.004; **Figure 3D**). Taken together, *C. sinensis* infection appears to mitigate secondary fungal risk through two parallel pathways: a direct biological pathway by elevating the host’s peripheral absolute lymphocyte count (mediating 12.11%), and an indirect clinical pathway by reducing the required duration of systemic corticosteroids exposure (mediating 10.91%).

## 4 Discussion

**Figure 4:**
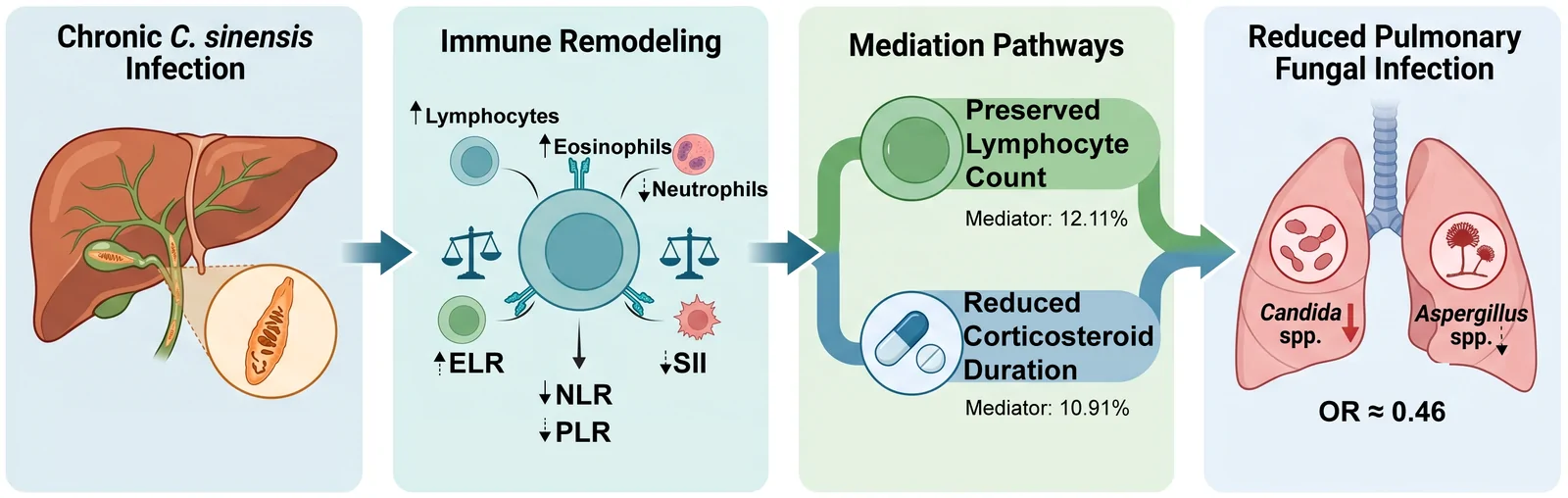
Hypothetical mechanistic model linking chronic *C. sinensis* infection to reduced pulmonary fungal infection (PFI) risk in AECOPD. *C. sinensis* infection may induce systemic immune remodeling to protect against secondary PFIs (overall OR ≈ 0.46). Mediation analyses suggest this association is partially mediated by preserving peripheral lymphocyte counts (mediating 12.11% of the effect) and reducing corticosteroid duration (10.91%). Because peripheral blood cell counts provide only indirect evidence, this model is speculative and serves as a hypothesis-generating framework for future research. *C. sinensis*, *Clonorchis sinensis*; ELR, eosinophil-to-lymphocyte ratio; NLR, neutrophil-to-lymphocyte ratio; PLR, platelet-to-lymphocyte ratio; SII, systemic immune-inflammation index; OR, odds ratio.

The present study provides the first clinical evidence that *Clonorchis sinensis* infection is independently associated with a reduced risk of pulmonary fungal infection in patients hospitalized for AECOPD. This association was consistently demonstrated across a hierarchical causal inference framework and remained robust after accounting for acute disease severity and strict diagnostic criteria. As proposed in our hypothetical model (**Figure 4**), helminth-associated immune remodeling may attenuate susceptibility to opportunistic fungal infections. Mediation analyses identified the peripheral absolute lymphocyte count and a reduced duration of systemic corticosteroids exposure as two parallel mediating pathways.

Patients with AECOPD exhibit marked impairment of cellular immunity, including reduced peripheral T cell counts and a decreased CD4^+^/CD8^+^ ratio (26, 27). Consistent with this, previous studies have shown that lymphopenia and dysfunction of adaptive immune responses are associated with increased susceptibility to opportunistic fungal infections in chronic respiratory diseases (28, 29). Experimental studies provide biological plausibility for our findings. Kim et al. demonstrated that *Clonorchis sinensis*-derived proteins can modulate airway immune responses through expansion of Tregs and induction of tolerogenic dendritic cell phenotypes (30). Similar immunomodulatory effects have also been reported in experimental schistosomiasis models (31). Together, these findings suggest that chronic helminth exposure may contribute to a more balanced immune environment, potentially influencing susceptibility to secondary pulmonary infections. While the expansion of these regulatory subsets conventionally counter-regulates Th1/Th17 pathways, they may also serve to attenuate excessive systemic inflammation during AECOPD, thereby contributing to the milder clinical trajectory observed in our cohort. Our mediation analysis identified the quantitative preservation of absolute lymphocyte count as a statistical mediator; however, whether this lymphocytosis reflects functionally preserved antifungal effector cells or an expanded regulatory pool requires further mechanistic investigation.

Interestingly, the incidence of both *Candida* spp. and *Aspergillus* spp. infections was numerically lower among patients with *C. sinensis* infection. However, statistical significance was observed only for *Candida* spp. infections, whereas the reduction in *Aspergillus* spp. infections did not reach significance. Several explanations may account for this finding. First, the relatively small number of *Aspergillus* infections in the matched cohort may have limited statistical power to detect moderate between-group differences. Second, distinct fungal pathogens may exhibit different sensitivities to alterations in host immune status. *Candida* spp. are common mucosal commensals whose pathogenic transition is closely linked to the integrity of adaptive immune responses and mucosal immune surveillance (32). In contrast, early pulmonary control of *Aspergillus* relies more heavily on innate immune mechanisms, including alveolar macrophages and neutrophils (33). Consistent with this interpretation, our mediation analyses identified peripheral lymphocyte count as a significant mediator of the association between *C. sinensis* infection and reduced fungal infection risk.

Although eosinophilia represented a prominent immunological feature among patients with *C. sinensis* infection, eosinophils contributed minimally to the observed protection against fungal infection. This finding suggests that eosinophilia may primarily represent a characteristic host response to helminth exposure rather than a direct mechanism of antifungal protection. Similarly, despite lower neutrophil proportions and reduced systemic inflammatory indices in the *C. sinensis* group, neutrophils did not significantly mediate the association. Taken together, these findings suggest that the observed association is unlikely to be explained solely by changes in individual innate immune cell populations. Our study only measured peripheral eosinophil counts, not their activation status or tissue infiltration levels. Therefore, we cannot completely exclude the role for eosinophils in antifungal defense at the mucosal level.

A parallel clinical mechanism identified in our study was the reduced duration of systemic corticosteroids exposure, which mediated 10.91% of the association. corticosteroids are pivotal in AECOPD management but are well-established risk factors for PFI. Importantly, because baseline hepatic function and other comorbidities were rigorously balanced via propensity score matching, and acute exacerbation severity was adjusted for in our sensitivity models, this reduced corticosteroid requirement is unlikely to be an artifact of conservative prescribing practices. Instead, it likely reflects an intrinsic moderation of the exacerbation in *C. sinensis* infected patients. Furthermore, the persistence of a robust association after comprehensive adjustment for clinical treatments suggests that intrinsic host immune factors operate concurrently with this clinical pathway.

Our findings also raise intriguing translational questions regarding the immunomodulatory properties of helminth-derived molecules. Increasing evidence indicates that parasite-derived antigens, excretory–secretory products, and metabolic mediators can exert potent immunoregulatory effects on host immune cells (34, 35). Although the present study was not designed to identify the specific molecular mediators involved, the observed association suggests that parasite-induced immune remodeling may have broader implications for host defense against secondary microbial pathogens. Future studies should focus on identifying the parasite-derived antigens, excretory–secretory products, and immunometabolic mediators responsible for this protective phenotype. Elucidation of these pathways may reveal novel host-directed immunotherapeutic strategies for preventing or treating opportunistic fungal infections in vulnerable populations.

Several limitations should be acknowledged. First, the retrospective single-center design carries an inherent risk of unmeasured confounding. Patients with *C. sinensis* infection may differ from uninfected controls in unmeasured characteristics, including socioeconomic status, dietary and environmental exposures, nutritional status, prior medication history, and healthcare-seeking behavior. Although propensity score matching and multivariable adjustment were performed to minimize confounding by measured baseline variables, residual confounding cannot be completely ruled out; however, our E-value quantifications suggest that such confounders would require a substantial effect size to nullify the primary findings. Second, to minimize the misclassification of fungal colonization, we validated our findings using a stringent outcome definition (≥ 7 days of systemic antifungal therapy); nevertheless, some diagnostic heterogeneity associated with retrospective clinical data may remain. Third, the specific burden and chronicity of *C. sinensis* infection could not be determined, precluding the assessment of dose-response relationships. Finally, because the absolute lymphocyte count only provides a quantitative measure, our study could not assess detailed T-cell functional quality or subset polarization. Future prospective multicenter studies incorporating comprehensive baseline data collection and serial functional immune monitoring are required to establish temporal relationships, minimize the impact of unmeasured confounding, and confirm the underlying biological mechanisms.

## 5 Conclusions

In conclusion, *C. sinensis* infection was independently associated with a substantially lower risk of pulmonary fungal infection in hospitalized patients with AECOPD. This association appeared to be partially mediated by preservation of peripheral lymphocyte counts and reduced systemic corticosteroids exposure. These findings provide novel clinical evidence linking helminth-associated immune regulation to susceptibility to opportunistic fungal infections. Future mechanistic studies integrating immunophenotyping, parasite-specific immune responses, and longitudinal clinical follow-up are needed to clarify the biological pathways underlying this association.

## Data Availability

The raw data supporting the conclusions of this article will be made available by the authors, without undue reservation.
